# Impact of COVID-19 on the Mode of Presentation and Stage at Diagnosis of Colorectal Cancer

**DOI:** 10.7759/cureus.32037

**Published:** 2022-11-30

**Authors:** Mahmoud Alrahawy, Charles Johnson, Medhet Aker, Hazim A Eltyeb, Susan Green

**Affiliations:** 1 Department of General Surgery, Menoufia University, Menoufia, EGY; 2 General and Colorectal Surgery, Durham University Hospital, Durham, GBR; 3 General and Colorectal Surgery, Colchester Hospital University, Colchester, GBR; 4 General Surgery, Health Education North East, Newcastle, GBR

**Keywords:** colorectal cancer (crc), covid-19, coronavirus, staging, emergency presentation

## Abstract

Aim: This study compares the stage at the presentation of patients with colorectal cancer (CRC) before and after introducing COVID-19 restrictions and the mode of presentation.

Methods: This is a retrospective cohort study comparing the incidence of CRC, TNM stage and mode of presentation in the pre-COVID and COVID cohorts at a single UK Trust. All patients discussed at the CRC multidisciplinary team (MDT) from March 2017 to March 2021 were included and split into two cohorts; the pre-COVID group from 01/03/2017 to 29/02/2020 and the COVID group from 01/03/2020 to 28/02/2021. Percentages were used for descriptive statistics. Student’s t-test was used for the comparison of demographic variables. Chi-squared test was used for the difference analysis for the categorical data, such as TNM and mode of presentation. P value ≤0.05 was significant.

Results: In total, 1373 patients were diagnosed with CRC during the period from March 2017 to March 2021. The pre-COVID group (2017-2020) included 1104 CRC patients, compared to 269 patients in the COVID one (2020-2021). The mean age was higher in the pre-COVID group (p = 0.001). There was a statistically significant increase in the proportion of cases presenting with T4 disease (p = 0.023) and metastatic disease (p = 0.032) in the COVID group compared to the pre-COVID group. There was also a significant increase in the rate of emergency presentations (p < 0.0001).

Conclusion: We observed a statistically significant increase in rates of locally advanced (T4) and metastatic (distant) CRC in patients presenting after introducing the COVID-19 lockdown. There was also an increase in emergency presentations. There was no observed difference in nodal status. This may reflect disruption to cancer diagnostic services and the reluctance of patients to access medical care during a pandemic, particularly the elderly.

## Introduction

Colorectal cancer (CRC) is the fourth commonest cancer in the United Kingdom but has the second lowest five-year survival rate, after lung cancer [1]. Early detection significantly improves the prognosis [2]. Thus, one of the NHS’s key ambitions in the long-term plan (published January 2019) is that by 2028, 75% of people with cancer will be diagnosed at an early stage (stage one or two) [3]. The introduction of the Rapid Diagnostic Centre (RDC) pathways and the Faster Diagnosis Standard (FDS) (reference) was necessary to improve cancer services. Patients are diagnosed or have cancer ruled out within 28 days of being referred urgently by their GP for suspected cancer, and this helps speed up cancer diagnosis and thus hopefully prognosis [4]. The established waiting times to start treatment-62 and 31-day pathways (62 days from the date the hospital receives an urgent referral for suspected cancer to the start of treatment and no more than 31 days wait between the consultation at which the treatment plan is agreed and start of treatment) still remain [5]. Surgical resection with curative intent is the gold standard for early stage CRC, with adjuvant or neo-adjuvant chemo-/radiotherapy for advanced stages [6].

Because of the exponential surge in COVID-19 (SARS-CoV-2) infections rate, the UK implemented a nationwide lockdown in March 2020, which affected the presentation and management of patients with CRC [7,8]. The potential risks of virus transmission during aerosol-generating procedures (e.g. endoscopy and laparoscopy) disrupted the screening services for CRC but also the diagnosis of symptomatic patients and subsequent surgical management and delivery of systemic chemotherapy and radiotherapy. The number of patients referred, diagnosed and treated for CRC decreased, resulting in missed diagnoses and more advanced stages at presentation [9,10]. Some units also abandoned laparoscopic procedures in favour of open surgery to avoid the potential risk of virus spread through smoke and CO_2_ deflation during laparoscopy [11].

The mode of presentation of CRC also impacts prognosis. Compared to elective surgery, emergency surgery for CRC is more likely to result in postoperative morbidity and death, particularly when the procedure is associated with obstruction or perforation [12]. This study aims to assess the impact of the COVID-19 pandemic on the mode of presentation of CRC and the stage at a presentation within our Trust. This article was previously presented as a meeting abstract at the 2020 European Society of Coloproctology (ESCP) ‘16th Annual Scientific Meeting online from 22 to 24 September 2020’.

## Materials and methods

This was a retrospective cohort study performed at a large, multi-site NHS Foundation Trust. We studied two groups of CRC patients, one who presented before the COVID-19 pandemic (Pre-COVID: from March 2017 to February 2020) and the second who presented during the pandemic (COVID group: from March 2020 to March 2021). To avoid an odd percentage in the pre-pandemic year, the pre-COVID group included data from three preceding years (March 2017- February 2020) to more accurately reflect our practice. Study approval was gained from the audit department in our Trust as a retrospective audit on practice.

All patients diagnosed with colonic or rectal adenocarcinoma between March 2017 and February 2021 were included. Patients’ data were identified retrospectively through the CRC multidisciplinary team (MDT) records. Data were collected from the Trust’s CRC dataset (Somerset Cancer Register). Patients with emergency or elective presentations were included, and the referral source was also identified. Any patient who needed to be evaluated by the on-call general surgery team was classified as an emergency presentation. Patient data were anonymized.

Statistical analysis was performed. Percentages were used for descriptive statistics. Student’s t-test was used for the comparison of demographic variables. Chi-squared test was used for the difference analysis for the categorical data, such as TNM staging and mode of presentation. The statistics software program used was Jeffreys’s Amazing Statistics Program (JASP 0.16.1 2021).

## Results

In total, 1373 patients were diagnosed with CRC during the period from March 2017 to March 2021. The pre-COVID group (2017-2020) included 1104 CRC patients, compared to 269 patients in the COVID one (2020-2021). The mean age was higher in the pre-COVID group (p = 0.001). There was also a 1.5-fold increase in the percentage of patients who presented in the emergency in 2020-2021 compared with 2017-2019 (p = 0.001) (Table 1).

**Table 1:** Table 1: CRC presentation pre- and during COVID-19 pandemic CRC: colorectal cancer

Characteristic	2017-2020 (n = 1104)	2020-2021 (n = 269)	P value
Age (ys) Mean(SD)	73.1 (11.9)	70 (12.4)	0.001
Stage T4 (%)	264 (24)	83 (31)	0.023
Node positive (%)	560 (51)	140 (52)	0.75
Metastasis (%)	197 (18)	64 (24)	0.032
Emergency (%)	147 (16)	22 (21)	<0.0001

**Figure 1:**
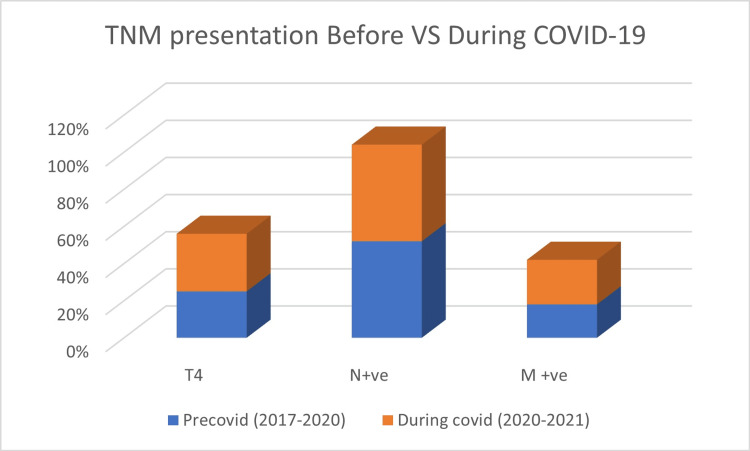
TNM presentation pre- and during COVID pandemic lockdown

There was a significant difference in the TNM tumour distribution between the COVID year and the precedent 3 years (Figure 1).

A 7% increase in T4 staging was shown between March 2020 and March 2021 compared with previous years with a p-value of 0.023 (Figure 1). There were more M1 cancers found during 2020-2021 than for 2017-2019 (24% vs. 18%, p = 0.032). Node-positive disease difference was insignificant in 2020 versus 2018-2019 (p = 0.75).

## Discussion

This study aimed to determine the influence of the COVID-19 pandemic on the stage and mode of presentation of patients with CRC at our Trust. Our study’s main conclusions were as follows: (1) the number of CRC patients that presented as an emergency increased during the COVID-19 pandemic (03/2020 to 02/2021) compared with the previous three years; (2) locally advanced (T4) and metastatic (M1) diseases’ percentages increased during the coronavirus widespread. Shinkwin et al. reported similar results with increasing numbers of CRC emergency presentations and T4 stages during the first wave of the 2020 lockdown [13]. They highlighted the importance of timely referral and early CRC detection to lower the cancer stage at presentation and prevent the necessity for emergency treatments with known worse outcomes, such as surgical site infections (SSI) and sepsis [14]. SSI is the most common postoperative complication after colorectal surgery, causing pain and suffering for patients. In addition, this complication has been associated with negative economic impact, increased morbidity, extended postoperative hospital stay, readmission, sepsis and death [15].

Large bowel blockage and intestinal perforation are the two most prevalent CRC presentations in emergencies [16]. Morbidity is almost tripled in emergent CRC surgeries compared to elective ones (40% and 9%), meanwhile, mortality is doubled (10% and 4%, respectively). In emergency CRC resections, the risk of postoperative complications (such as anastomotic leakage, permanent stoma creation) increases and pneumonia is especially common after surgeries for intestinal perforation. Relative risks of distant spread and poor survival outcomes are higher in emergent CRC procedures and obstructing tumours as well [17].

During the pandemic, endoscopic CRC screening was disrupted because of cancellation and patient concerns apprehension over getting COVID-19. These service interruptions have resulted in delayed and advanced presentations of CRC patients with the potentially poorer clinical outcome than usual standards. We have no long-term follow-up yet to look at the effects of the pandemic on the long-term survival of patients with CRC; however, management of CRC in more advanced stage results in a poorer prognosis, as cited in many previous studies [18,19].

The COVID-19 pandemic reduced emergency surgical operations and overall admissions to emergency departments because of the widespread hospital fear and anxiety experienced by most patients during the peak of this outbreak. The causes of the sharp reduction of emergency surgical cases during the COVID-19 pandemic are multi-factorial, reflecting a dangerous situation with serious consequences for public health. According to the results of Mulita et al., patients in the COVID era presented to the hospital with delayed onset of symptoms, in comparison with those admitted before the COVID pandemic, because of their anxiety and fear of being infected with the coronavirus. As a result the hospital stay, as well as the operation duration of patients in the COVID era, was increased [20].

We can learn lessons from the COVID-19 pandemic that will hopefully stand us in good stead for any future pandemics. Encourage patients to continue presenting to primary care with worrying symptoms in a timely fashion with support from public health campaigns and to ring-fence cancer services and diagnostics to prevent unnecessary delays.

Developing resilient primary health care is mandatory for better management of future pandemics. We can use new policies in frontline health services such as reshaping the roles of community health professionals and giving them more room to deal with chronic patients (e.g. pharmacist prescription of regular medications). Community facilities also need to be equipped with innovative digital technology that delivers virtual care that could encourage the vulnerable population to present early and avoid any risky delays [21].

The limitation of our study is that retrospective study that represents the COVID-19 effect on cancer service in a single NHS Trust. We have also not included survival analysis for the patients who presented after the coronavirus pandemic to examine the direct effect of a pandemic on CRC long-term oncological outcomes. Further multicentric trials with prolonged follow-up periods are mandatory to calculate these outcomes accurately.

## Conclusions

This study reported a significant rise in CRC emergency presentations, locally advanced (T4) and metastatic (remote) disease for patients who presented during the COVID-19 pandemic compared to those who presented pre-pandemic. The reported effects are most likely because of a pandemic’s impact on cancer diagnostic services and change in treatment pathways, but also a change in patients’ behaviour with a reluctance to seek medical care, especially the elderly.

In pandemics similar to coronavirus, new public health strategies are required to primarily protect cancer services from unnecessary delays that result in advanced and emergent CRC presentations. People need more motivation to continue attending primary care once they express any concerning symptoms.
